# Isolation and characterization of adenoviruses infecting endangered golden snub-nosed monkeys (*Rhinopithecus roxellana*)

**DOI:** 10.1186/s12985-016-0648-6

**Published:** 2016-11-25

**Authors:** Bing Tan, Li-Jun Wu, Xing-Lou Yang, Bei Li, Wei Zhang, Yong-Song Lei, Yong Li, Guo-Xiang Yang, Jing Chen, Guang Chen, Han-Zhong Wang, Zheng-Li Shi

**Affiliations:** 1 Key Laboratory of Special Pathogens and Center for Emerging Infectious Diseases, Wuhan Institute of Virology, Chinese Academy of Sciences, Wuhan, China; 2 Monitoring Center of Wildlife Diseases and Resource of Hubei Province, Wuhan, China; 3 University of Chinese Academy of Sciences, Beijing, China; 4 Present Address: Key Laboratory of Special Pathogens and Biosafety, Wuhan Institute of Virology, Chinese Academy of Sciences, Wuhan, 430071 China

**Keywords:** Mastadenovirus, Simian adenovirus, Golden snub-nosed monkey

## Abstract

**Background:**

Adenoviruses are important pathogens with the potential for interspecies transmission between humans and non-human primates. Although many adenoviruses have been identified in monkeys, the knowledge of these viruses from the *Colobinae* members is quite limited.

**Findings:**

We conducted a surveillance of viral infection in endangered golden snub-nosed monkeys (*Rhinopithecus roxellana*) in the subfamily *Colobinae* in China, and found that 5.1% of sampled individuals were positive for adenovirus. One of the adenoviruses (SAdV-WIV19) was successfully isolated and its full-length genome was sequenced. The full-length genome of WIV19 is 33,562 bp in size, has a G + C content of 56.2%, and encodes 35 putative genes. Sequence analysis revealed that this virus represents a novel species in the genus *Mastadenovirus*. Diverse cell lines, including those of human origin, were susceptible to WIV19.

**Conclusion:**

We report the first time the isolation and full-length genomic characterization of an adenovirus from the subfamily *Colobinae*.

**Electronic supplementary material:**

The online version of this article (doi:10.1186/s12985-016-0648-6) contains supplementary material, which is available to authorized users.

## Body of text

Within the family *Adenoviridae*, the genus *Mastadenovirus* contains a group of non-enveloped icosahedral viruses that range in size from 70 to 90 nm and contain a linear double-stranded DNA genome of approximate 35 kb [1]. Members of this genus are pathogens that infect a wide range of mammals. In humans, adenoviruses (AdVs) cause a variety of pathologies including acute respiratory illness, epidemic keratoconjunctivitis, acute haemorrhagic cystitis, hepatitis, myocarditis, and gastroenteritis [2]. Although usually self-limiting, AdV infection may induce serious morbidity and mortality, especially in immunocompromised patients and transplant recipients [3]. Similarly, AdVs have been associated with diarrhea, acute respiratory illness, pneumonia, and hepatitis in captive non-human primates [4–6]. In 2009, an outbreak of AdV-infection led to 83% fatality in titi monkeys of the genus *Callicebus*, a group of New World monkeys [6]. Notably, neutralizing antibodies against this titi monkey mastadenovirus were detected in two human individuals, indicating the potential for zoonotic transmission. Several other cases of potential AdV transmission between humans and non-human primates have also been documented recently [7–9].

To date, at least 40 distinct types of simian adenoviruses (SAdVs) have been reported. Most of them infect captive great apes and members of the subfamily *Cercopithecinae* [10, 11]. SAdVs that infect great apes are closely related to types that infect humans, which belong to the species *Human mastadenovirus A* to *Human mastadenovirus F* (HAdV-A-F) (primarily B, C, and E) [8, 11]; those infecting the *Cercopithecinae* members have been classified into HAdV-G, species *Simian mastadenovirus A* (SAdV-A), and the recently proposed species SAdV-B-H [1, 12, 13]. It should be noted that the group of Old World monkeys (OWMs) comprises two subfamilies, the *Cercopithecinae* and the *Colobinae*; however, knowledge of AdVs from the latter sub-group remains quite limited. Only a few short sequences of AdV DNA have been reported in this subfamily [11].

Golden snub-nosed monkeys (*Rhinopithecus roxellana*) living in Shennongjia Nature Reserve (SNR) in Hubei, China, are an endangered species belonging to the subfamily *Colobinae* [14]. Visitors to SNR have close contact with monkeys, which are fed by animal nurses in the reserve area; this raises the possibility of viral transmission between humans and monkeys. However, despite the extensive efforts to protect these monkeys from being endangered, viruses infecting these animals are poorly studied.

**Fig. 1:**
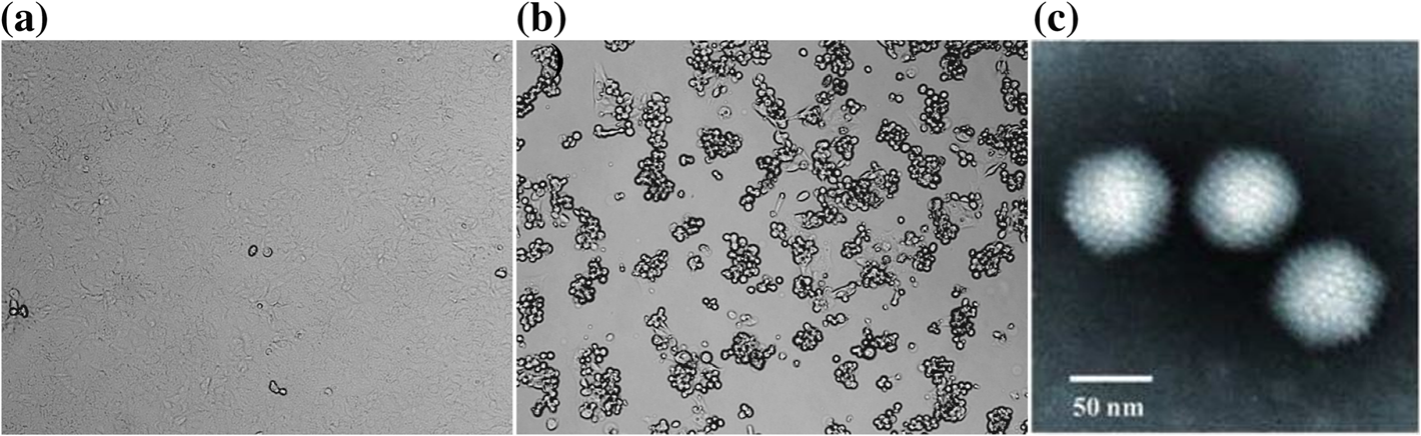
Isolation of a simian adenovirus. **a** Uninfected Vero E6 cells. **b** Infected Vero E6 cells (second passage) inoculated with an adenovirus-positive sample at 72 h post infection. **c** Electron microscopy of purified adenoviral particles

In this study, we conducted a surveillance of viral infections in 59 faecal samples from *R. roxellana* collected from SNR in 2014. Pan-PCR analysis was performed to detect the presence of AdVs, coronaviruses, enteroviruses, mammalian reoviruses, and rhinoviruses [15–17]. Except the 3 samples that tested positive for AdV, all samples were negative for the tested viruses. The virus strain in the 3 positive samples shared 100% nucleotide identity, based on the 261-bp DNA polymerase gene sequences. One of them was successfully isolated and cultured in Vero E6 cells (Fig. 1) and its full-length genome was sequenced. Following the order of viruses isolated in our laboratory at Wuhan Institute of Virology (WIV), we tentatively named this isolate as simian adenovirus WIV19 (SAdV WIV19).

**Fig. 2:**
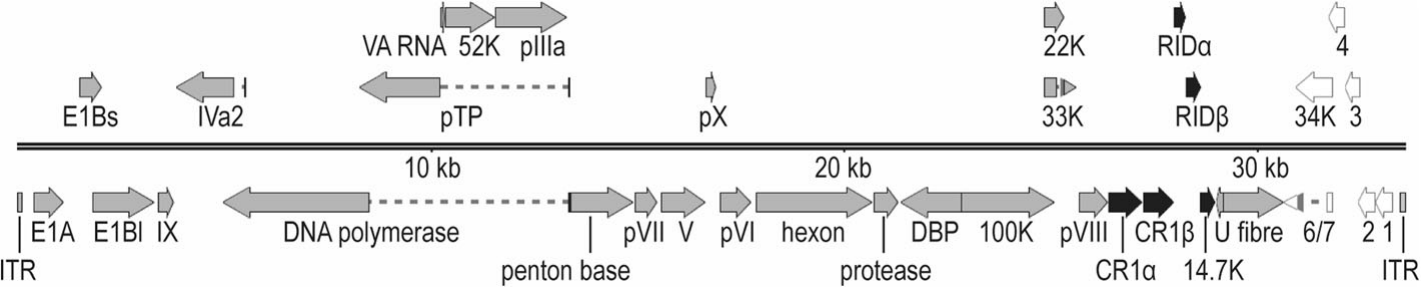
Genomic characterization of SAdV-WIV19. The genome of SAdV-WIV19 is represented by the double line, with the scale indicated in the center. Viral genes and ITR sequences are shown as arrows and rectangles, respectively. Coding regions linked with broken lines indicate predicted spliced transcripts. The E3 and E4 genes are shown in black and white, respectively. With the exception of the 34K gene, the E4 genes are indicated by numbers of the corresponding open reading frames. Note that the genomic organization of WIV19 is similar to that shared by SAdV-A and G members, except that WIV19 lacks the E3 12.5K gene

The WIV19 genome comprises 33,562 bp, including 22.10% A, 28.31% C, 27.92% G, and 21.67% T (GenBank accession number KX505867) (Fig. 2). Its 95-bp extreme ends are inversely repeated, and start with the conserved motif CATCATCAAT [18]. The genome was predicted to encode 34 proteins and a virus-associated RNA (Fig. 2). Although some primate AdVs are known to encode two fibre genes, only one copy was identified in the genome of WIV19. Similarly, a single gene of non-coding virus-associated RNA was identified between the second exon of pTP and the 52K gene. Except for the absence of the E3 12.5K gene, organization of the WIV19 genome was identical to that of SAdV-A and G isolates infecting OWMs.

The putative gene products of the WIV19 genome displayed 22–91% amino acid (aa) identities to their closest homologues encoded by members of SAdV-A (Additional file 1: Table S1). Most genes displayed >85% aa identity with those encoding structural proteins, including pIIIa, penton base, pVII, pVI, pVIII, and hexon. By contrast, fibre protein showed only 32% aa identity to its closest homologue, which may suggest distinct infectivity in vivo due to its critical role in host cell binding [19]. DNA polymerase is usually used for classification of AdV [1]. Based on analysis of sequences of this gene, WIV19 displayed the highest aa identity (77%) to SAdV-3, suggesting that it is a distinct species.

**Fig. 3:**
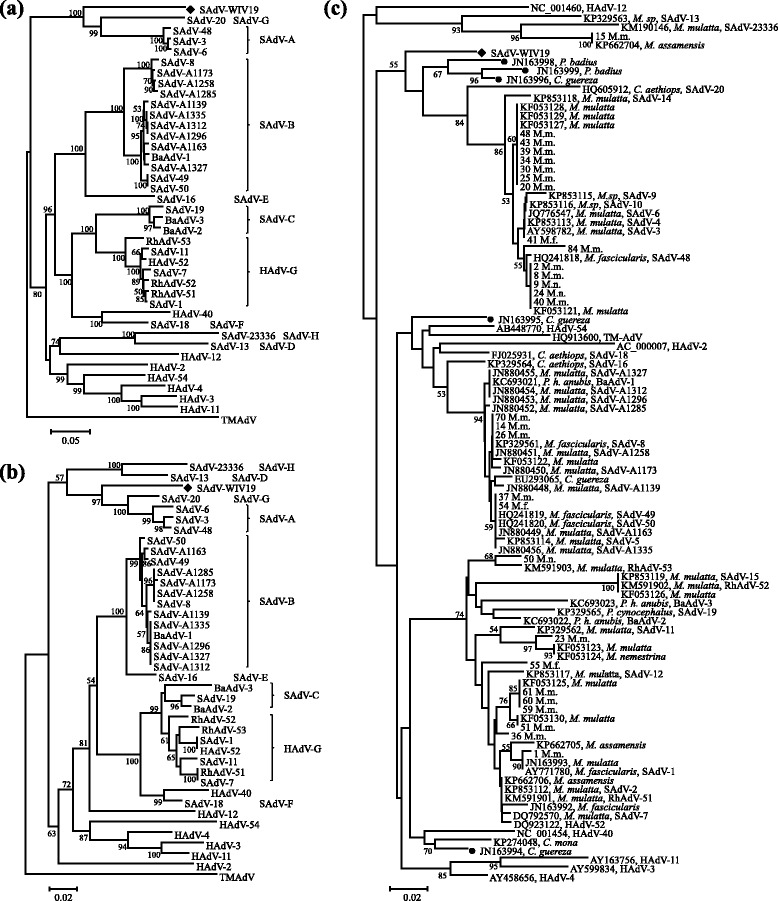
Phylogenetic analysis based on the full-length amino acid sequences of DNA polymerase (**a**) and penton base (**b**) and partial nucleotide sequences of the *hexon* genes (**c**). Diamonds indicate the virus isolated in this study. Dots indicate adenoviruses from the subfamily *Colobinae*. HAdV, human adenovirus; SAdV, simian adenovirus; TMAdV, titi monkey adenovirus. In the partial *hexon* tree, adenoviruses infecting Old World monkeys are indicated by the GenBank accession numbers, followed by host species and type names (selected). “XX M.f.,” “XX M.m.,” and “XX M.n.” (XX = sample number) indicate Chinese strains identified by Banyai, K., et al.; and M.m., M.f., and M.n. indicate the host species of *Maccaca mulatta*, *M. fascicularis*, and *M. nemestrina*, respectively [25]. *C. mona, Cercopithecus mona; C. aethiops, Chlorocebus aethiops; C. guereza, Colobus guereza; P. cynocephalus, Papio cynocephalus; P. h. anubis, Papio hamadryas anubis; P. badius, Piliocolobus badius.* The phylogenetic trees were constructed using the program MEGA, version 6.0, using the neighbor-joining method with 1,000 bootstrap replicates. Percentage bootstrap values >50 are indicated at the nodes. Scale bars indicate evolutionary distance

Comparison of protein sequence similarities suggested that WIV19 is distantly related to OWM AdVs. Phylogenetic trees were constructed for better evaluation of the evolutionary relationship between WIV19 and the reported AdVs. Consistently, WIV19 formed a separate branch closer to a common ancestor of SAdV-A and G based on analyses of both DNA polymerase and penton base sequences (Fig. 3a, b). When we used the partial *hexon* sequences available in all OWM types, a common ancestor of three *Colobinae* types (JN163998, JN163999, and JN163996) was identified as the nearest neighbor to WIV19 (Fig. 3c). These observations suggested coevolution between WIV19 and golden snub-nosed monkeys.

Homologous recombination has been demonstrated to be an important evolutionary mechanism for AdVs, especially for HAdV-D members [20]. However, no signals of recombination were identified in the WIV19 genome, using RPD4 and SimPlot software [21, 22].

For investigating the antibody prevalence against the AdV infection, virus neutralization assay was performed using archived serum samples collected from 8 golden snub-nosed monkeys in SNR [23]. As a result, cross-neutralizing antibodies against WIV19 were identified in all the 8 samples at titers ranging from 40 to 160.

The potential host range of WIV19 was evaluated using cell infectivity assays. Seven cell lines were tested: A549 (human lung, ATCC CRM-CCL-185), HEK-293 T (human embryonic kidney, ATCC CRL-11268), NCI-H292 (human lung, ATCC CRL-1848), LLC-MK2 (*Macaca mulatta* kidney, ATCC CCL-7), MsIn (*Miniopterus schreibersi* intestine), RlKi (*Rousettus leschenaulti* kidney), and RsKi (*Rhinolophus sinicus* kidney) [24]. Cytopathic effects were observed in LLC-MK2, RsKi, and all three human cell lines, indicating that WIV19 has a wide host range.

In conclusion, we described here the first isolation and characterization of a replication-competent AdV from monkeys in the subfamily *Colobinae*. The cumulative prevalence of AdV in endangered golden snub-nosed monkeys from SNR was found to be 5.1%. The present isolate from these monkeys is distantly related to known AdV types, and likely represents a novel species in the genus *Mastadenovirus*. Its ability to infect diverse cell lines, including those of human origin, highlights the potential risk of new and emerging infections in tourists visiting SNR. Result from the virus neutralization assay suggested that the infections of WIV19 may be highly prevalent in these monkeys. Further studies are required to determine its host range and role in causing disease in both golden snub-nosed monkeys and humans. Long-term surveillance of viral prevalence in this region should be conducted in the future.

## Additional file

Additional file 1: Table S1. Predicted genes of WIV19 and comparison to those of known adenoviruses. (DOCX 20 kb)

## Abbreviations

AdVs: Adenoviruses
HAdV: Human mastadenovirus
OWMs: Old World monkeys
SAdV: Simian adenovirus or mastadenovirus
SNR: Shennongjia Nature Reserve
WIV: Wuhan Institute of Virology
