# Supplementary material for: Isolation and characterization of adenoviruses infecting endangered golden snub-nosed monkeys (Rhinopithecus roxellana)
Source: Virol J. 2016 Nov 25;13:190. doi: 10.1186/s12985-016-0648-6 (PMC5123214; doi:10.1186/s12985-016-0648-6)
Supplement: Additional file 1: Table S1. — Predicted genes of WIV19 and comparison to those of known adenoviruses. (DOCX 20 kb) [file 12985_2016_648_MOESM1_ESM.docx]

**Supplementary Materials**

**Isolation and characterization of adenoviruses infecting endangered golden snub-nosed monkeys (*Rhinopithecus roxellana*)**

Bing Tan^1,3^, Li-Jun Wu^1^, Xing-Lou Yang^1^, Bei Li^1^, Wei Zhang^1^, Yong-Song Lei^2^, Yong Li^2^, Guo-Xiang Yang^2^, Jing Chen^2^, Guang Chen^2^, Han-Zhong Wang^1^, Zheng-Li Shi^1,3^#

^1^Key Laboratory of Special Pathogens and Center for Emerging Infectious Diseases, Wuhan Institute of Virology, Chinese Academy of Sciences, Wuhan, China;

^2^Monitoring Center of Wildlife Diseases and Resource of Hubei Province, Wuhan, China;

^3^University of Chinese Academy of Sciences, Beijing, China.

Running head: Adenoviruses infecting golden snub-nosed monkeys

# Address correspondence to Zheng-Li Shi: Key Laboratory of Special Pathogens and Biosafety, Wuhan Institute of Virology, Chinese Academy of Sciences, Wuhan 430071, China; Tel: +86 27 87197240; Email address: [zlshi@wh.iov.cn](mailto:zlshi@wh.iov.cn)

Supplementary Table Predicted genes of WIV19 and comparison to those of known adenoviruses.

| Gene name | Location (s) (nt) | Size (aa) | Closest homologues (aa identity, length) |
| --- | --- | --- | --- |
|  |  |  |  |
| ITR | 1..95 | 95 bp | SAdV-3 (65% [nt], 173 bp) |
| E1A | 407..1141 | 244 | SAdV-20 (39%, 267 aa) |
| E1Bs | 1516.. 2058 | 180 | SAdV-48 (67%, 184 aa) |
| E1Bl | 1821.. 3332 | 503 | SAdV-20 (70%, 506 aa) |
| IX* | 3414.. 3806 | 130 | SAdV-20 (68%, 136 aa) |
| IVa2* | 3864..5218, 5497..5509c | 455 | SAdV-48 (81%, 454 aa) |
| DNA polymerase | 4988..8497, 13341..13349c | 1172 | SAdV-3 (77%, 1172 aa) |
| pTP* | 8299..10218, 13341..13349c | 642 | SAdV-6 (83%, 649 aa) |
| VA RNA | 10248..10341 | 94 bp | SAdV-3 (61% [nt], 99 bp) |
| 52K | 10357..11559 | 400 | SAdV-3 (85%, 396 aa) |
| pIIIa* | 11577..13319 | 580 | SAdV-48 (88%, 579 aa) |
| penton base* | 13397..14932 | 511 | SAdV-3 (86%, 504 aa) |
| pVII* | 14946..15503 | 185 | SAdV-6 (88%, 185 aa) |
| V* | 15573..16673 | 366 | SAdV-3 (70%, 356 aa ) |
| pX* | 16673..16924 | 83 | SAdV-3 (81%, 73 aa) |
| pVI* | 17000..17773 | 257 | SAdV-3 (85%, 256 aa) |
| hexon* | 17871..20708 | 945 | SAdV-3 (87%, 932 aa) |
| protease* | 20725..21348 | 207 | SAdV-6 (77%, 202 aa) |
| DBP | 21391..22839c | 482 | SAdV-3 (64%, 466 aa) |
| 100K | 22854..25124 | 756 | SAdV-3 (72%, 732 aa) |
| 33K | 24862..25150, 25326..25642 | 201 | SAdV-3 (49%, 178 aa) |
| 22K | 24862..25344 | 160 | SAdV-3 (49%, 147 aa) |
| pVIII* | 25694..26398 | 234 | SAdV-3 (91%, 232 aa) |
| E3 CR1α | 26414..27232 | 272 | SAdV-3 (22%, 369 aa) |
| E3 CR1β | 27257..27994 | 245 | SAdV-48 (30%, 263 aa) |
| E3 RIDα | 27985..28287 | 100 | SAdV-48 (57%, 91 aa) |
| E3 RIDβ | 28284..28643 | 119 | SAdV-3 (37%, 105 aa) |
| E3 14.7K | 28633..29004 | 123 | SAdV-23336 (66%, 127 aa) |
| U exon | 29008..29169c | 53 | SAdV-6 (55%, 54 aa) |
| fibre* | 29178..30647 | 489 | SAdV-48 (33%, 545 aa) |
| E4 ORF6/7 | 30669..30965, 31682..31813c | 142 | SAdV-48 (58%, 150 aa) |
| E4 34K | 30962..31813c | 283 | SAdV-3 (77%, 284 aa) |
| E4 ORF4 | 31743..32108c | 121 | SAdV-3 (76%, 121 aa) |
| E4 ORF3 | 32124..32471c | 115 | SAdV-6 (64%, 115 aa) |
| E4 ORF2 | 32443..32859c | 138 | SAdV-3 (62%, 129 aa) |
| E4 ORF1 | 32873..33259c | 128 | SAdV-6 (67%, 128 aa) |
| ITR | 33468..33562 | 95 bp | SAdV-3 (65% [nt], 173 bp) |

nt, nucleotide; aa, amino acid; *, structural protrein; c, encoded product on the complementary strand; SAdV, simian mastadenovirus.
